# The Arts of the Theatre at Fulham Infirmary

**Published:** 1913-01-18

**Authors:** 


### THE ARTS OF THE THEATRE AT FULHAM INFIRMARY.

### A Remarkable Example of Rehearsing and Staging.

Eleven o'clock on Friday night last week in the corridor of Fulham Infirmary; an excited crowd talking and laughing and struggling for refreshments. But in one corner the laughter is merrier, the excitement greater, the talk crisper and quicker. For this is a group of the old sisters and probationers revisiting their old school: laudators of times past but full of delighted praises of the performance of the present. "Oh, Mac, wasn't it great?" "Don't you remember when we were Irish colleens and Highland lasses?" "Jones, come and talk to me." "There's little Pye," and so on, and so on. The event was the annual entertainment given by the infirmary nursing staff. On the previous night the performance had been given to all those patients who could be brought by any means to the ward in which the stage had been erected. Now on this night the performance had been repeated to an audience of nurses' friends, former members of the medical and nursing staffs, and the Guardians. And what a performance it was! The present equalled and in some respects surpassed all previous records.

The scene on the stage for the first part of the programme represented a little village of wooden chalets, overgrown with flowers and dominated by a massive snow-clad mountain rising from moss-grown slopes sprinkled with gay flowers. A cunning system of lighting gave depth and atmosphere to the scene. As the curtain rose gaily-dressed country girls and boys strolled on, and arranging themselves into picturesque groups, broke into a song of welcome to the Spring. One extremely effective scene was that in which, with the stage in complete darkness save for the lights gleaming in the windows of the chalets and mill of the village, and for the flashes of tiny moving electric lamps shining like glow-worms at the foot of the mountain and in her hair, Nurse Fail sang, with great sweetness and skill, "Shine, little Glow-worm, shine on." Following this, Nurse Kyle, in Highland costume, sang "The Wee Traveller" with vivacity and a Scotch accent which evoked delighted applause.

Between the two parts of the performance two short plays were given. Influenced by the "miniature method" as exemplified in the recent production at the Court Theatre of Tolstoi's "The Man who was Dead," under the direction of M. Andreev, of the Royal Theatre, Belgrade, a tiny scene representing the whitewashed interior of an Irish cottage was built up in the centre of the stage. In this setting a short miracle-play entitled "The Wanderer" was given. The part of the mother was taken by Sister Mason, that of the child by Nurse Hogan, and the very difficult and trying part of the Wanderer by Nurse Hayes. The naturalness of the acting of Sister Mason and Nurse Hogan in their respective parts, contrasted with the solemn and beautiful rendering of her part by Nurse Hayes, created a great effect. This play was followed by an Irish farce, "The Magic Stone," acted by Nurses Burke, O'Riordan, and Ryan. The inimitable Irish brogue of these three nurses, the witty dialogue, the droll gestures, the vivacity, artistic finish, and sparkle of the acting, and the departure from stage convention by which those parts of the dialogue which were supposed to be heard off the scene were acted in the audience itself caused roars of laughter.

For the second part of the programme the same scene was retained as in the first part. But spring had changed to winter, the flowers were replaced by snow and icicles, and the green slopes of the mountain by snowfields, gleaming in the cold blue of the gathering night and the red glow from the lights of the cottages. A further selection of songs was given by the chorus, now dressed in the bright, warm costumes affected by visitors to Swiss winter resorts. A solo, "Life is so sweet," was charmingly rendered by Nurse Ryan. Nurse Comerford sang "Snow" with great animation, accompanying the chorus of the song with a pretty dance, the general effect being heightened by a realistic fall of snow on the slopes of the mountain in the background. A duet entitled "Later on—perhaps," amusingly sung by Nurses Kyle and Siviour, caused much laughter. A farcical operetta, "Domestic Economy," in which the part of the spendthrift wife was taken by Nurse Fail and that of her husband by Nurse Ryan, was acted and sung in a manner which provoked roars of merriment. Nurse Mills gave a capital recitation, "Her First Cigarette." An unrehearsed effect was produced in the middle of her performance by the blowing of a fuse, which plunged the whole stage in pitchy darkness. Nurse Mills, however, kept her head better than the stage manager, who nearly exploded, and went on calmly, and the audience, which evidently regarded it as symbolic of her failure to light a match, laughed in sympathy. Nurse Watson was heartily applauded for a violin solo, "Simple Aveu," rendered with great tenderness and technical finish. Two dances—arranged by Miss Heather Nepean—a gavotte and a minuet, were most gracefully and charmingly stepped by Sister Mason and Nurses Evans, Green, Comerford, Kyle, and Sullivan. The effect was enhanced by the beautiful dresses, designed, fitted, and made by Nurse Hayes. The dress of the gentlemen consisted of mauve or pink full-skirted silk coats, the fronts trimmed with narrow silk black braid and the cuffs with braid and deep lace ruffles, and knee breeches of pale green silk. The ladies carried ostrich-feather fans, and wore cream skirts with panniers of blue or pink silk, short-sleeved bodices to match, trimmed with a fichu and frills of white chiffon and a large pink rose, white underskirts trimmed with blue ribbon, lace, and tiny roses, mittens, and buckled shoes, and a black band of velvet round the neck. At the end of the minuet loud cries were raised for Miss Nepean, and when she appeared on the stage in answer she was presented with a beautiful basket of flowers by the dancers. During the performance Dr. Swindells sang several comic songs in a manner which deservedly earned many recalls. He was accompanied by Mrs. Huth and Miss Gibbs. The accompaniment of the other songs and choruses was undertaken by Nurse Boileau, who also worked indefatigably at the numerous rehearsals.

The heavy work of organising the rehearsals and the performances fell upon the capable shoulders of the Matron, Miss Ballantyne, and of the Assistant Matron, Miss Bevan. Part of the scenery was very kindly lent by Mr. Wentworth Croke, of the Lyric Theatre, Hammersmith. The stage and the rest of the scenery was skilfully constructed by Mr. Hull, the works foreman, and the electrical effects were arranged by Mr. Sharpe, the infirmary engineer. In addition to the dresses for the dancers Nurse Hayes also designed and made the dresses for the choruses. The parts in the chorus were taken by Nurse Comerford, Nurse Davies, Nurse Evans, Nurse Fail, Nurse Green, Nurse Hogan, Nurse Jenkins, Nurse Kyle, Nurse Nightingale, Nurse Ryan, Nurse Siviour, and Nurse Watson.

